# Whole Genome Sequence-Based Analysis of Bovine Gammaherpesvirus 4 Isolated from Bovine Abortions

**DOI:** 10.3390/v16050739

**Published:** 2024-05-08

**Authors:** Florencia Romeo, Maximiliano Joaquín Spetter, Susana Beatriz Pereyra, Pedro Edgardo Morán, Erika Analía González Altamiranda, Enrique Leopoldo Louge Uriarte, Anselmo Carlos Odeón, Sandra Elizabeth Pérez, Andrea Elizabeth Verna

**Affiliations:** 1 Instituto Nacional de Tecnología Agropecuaria, Instituto de Innovación para la Producción Agropecuaria y El Desarrollo Sostenible (IPADS, INTA-CONICET) Ruta 226, km 73.5, Balcarce CC7620, Buenos Aires, Argentinaelougeuriarte@gmail.com (E.L.L.U.); 2 Facultad de Ciencias Veterinarias, Departamento de Fisiopatología, Centro de Investigación Veterinaria de Tandil (CIVETAN), Universidad Nacional del Centro de la Provincia de Buenos Aires, Paraje Arroyo Seco s/n, Tandil CC7000, Buenos Aires, Argentina; 3 Laboratorio de Virología, Facultad de Ciencias Veterinarias, Centro de Investigación Veterinaria de Tandil (CIVETAN), Universidad Nacional del Centro de la Provincia de Buenos Aires, Paraje Arroyo Seco s/n, Tandil CC7000, Buenos Aires, Argentina; 4 Facultad de Ciencias Agrarias, Universidad Nacional de Mar del Plata, Ruta 226, km 73.5, Balcarce CC7620, Buenos Aires, Argentina

**Keywords:** bovine gammaherpesvirus 4, Argentinean strains, genome analysis

## Abstract

Bovine gammaherpesvirus 4 (BoGHV4) is a member of the *Gammaherspivirinae* subfamily, *Rhadinovirus* genus. Its natural host is the bovine, and it is prevalent among the global cattle population. Although the complete genome of BoGHV4 has been successfully sequenced, the functions of most of its genes remain unknown. Currently, only six strains of BoGHV4, all belonging to Genotype 1, have been sequenced. This is the first report of the nearly complete genome of Argentinean BoGHV4 strains isolated from clinical cases of abortion, representing the first BoGHV4 Genotype 2 and 3 genomes described in the literature. Both Argentinean isolates presented the highest nt p-distance values, indicating a greater level of divergence. Overall, the considerable diversity observed in the complete genomes and open reading frames underscores the distinctiveness of both Argentinean isolates compared to the existing BoGHV4 genomes. These findings support previous studies that categorized the Argentinean BoGHV4 strains 07-435 and 10-154 as Genotypes 3 and 2, respectively. The inclusion of these sequences represents a significant expansion to the currently limited pool of BoGHV4 genomes while providing an important basis to increase the knowledge of local isolates.

## 1. Introduction

Bovine gammaherpesvirus 4 (BoGHV4) is a member of the *Gammaherpesvirinae* subfamily, *Rhadinovirus* genus [1]. Unlike other rhadinoviruses, BoGHV4 has demonstrated the capability to replicate in a wide range of species [2] as well as across a broad variety of cell cultures, including animal and human cell lines and primary cultures [2,3,4,5]. Its natural host is the bovine, and it is prevalent among the global cattle population. Despite the identification of BoGHV4 in animals displaying asymptomatic infection as well as in animals showing a variety of clinical signs [3,6], the pathogenic role of BoGHV4 remains unclear, not being recognized as an etiological agent of a particular pathology.

The genome of BoGHV4 comprises a double-stranded DNA of ~144 kb in length. The viral genome is composed of a core region (long single coding region or LUR) of ~108 kb, with low G + C content and flanked at both ends by high G + C multiple repeats called polyrepeat DNA (prDNA) [7]. The viral genome contains at least 79 open reading frames (ORFs), with 17 of them being specific for BoGHV4 (Bo1 to Bo17) [7].

As in other herpesviruses, the BoGHV4 genome is enclosed and protected by an icosahedral capsid, which includes 162 capsomeres [8]. The envelope is composed of approximately a dozen viral proteins and glycoproteins anchored in a lipid bilayer.

The main glycoproteins present in BoGHV4 are gB, gH, gL, gp180, and gM [9], three of which are essential for the cell entry of herpesviruses: gB, gH, and gL. Glycoprotein gp180 plays a crucial role in the interaction between the virus and host cells. Specifically, it is involved in the binding of the virus to cellular receptors, a critical step in the viral infection process [10,11]. Understanding the role of BoGHV4 gp180 in virus binding and tropism is crucial for unraveling the mechanisms of BoGHV4 infection and pathogenesis. This knowledge may contribute to the development of strategies to control or prevent BoGHV4 infections, potentially aiding in the design of specific antiviral therapies or vaccines.

The BoGHV4 genome possesses a limited number of ORFs, which are like cellular genes. One of these is the Bo17 gene, responsible for encoding ß-1,6-N-acetylglucosaminyltransferase (ß-1,6 GnT), which shares 81.1% similarity with the human enzyme [12]. This gene was acquired from an African buffalo ancestor and, while not crucial for virus replication, plays a role in the post-translation modification of virion proteins by adding glycans [13]. This process may lead to alterations in viral tropism or susceptibility to neutralization by antibodies or the complement system, promoting the virus evasion of the immune response [13].

Bovine gammaherpesvirus 4 presents genomic variations that have been demonstrated in genetic studies of reference strains from the American (DN599 and 66-p-347) and European (V. test, LVR140 and Movar) groups. These studies identified important differences in at least nine ORFs, along with variations in the length of prDNA segments [7,14,15,16]. Furthermore, variability has been observed among isolates from different regions and the emergence of new strains, whose restriction patterns do not fully correspond to the previously mentioned prototypes [16,17].

In Argentina, BoGHV4 was detected in 2007 from cows with a history of abortion [18]. Since then, it has been isolated in different regions of the country from cervicovaginal mucus [19], brain tissue [20], semen from an artificial insemination center, nasal swabs, oocytes and granulosa cells [21], and fetal tissues [22].

Verna et al. [18] demonstrated a high genetic variability of Argentinean BoGHV4 strains isolated from vaginal fluids of aborted cows and the existence of a new phylogenetic group known as Genotype 3. Romeo et al. [23,24] and Verna et al. [25] conducted comparative studies of two Argentinean BoGHV4 strains corresponding to Genotypes 2 and 3 (10-154 and 07-435 strains, respectively), showing significant molecular and biological distinctions between these isolates. A noteworthy finding is the feature of strain 07-435 to be neutralized by sera from various ruminant species [23], highlighting a distinctive trait of this strain. Supporting this, Dubuisson et al. [26] demonstrated in their research that the humoral immune response in bovines infected with BoGHV4 is characterized by low efficiency or low levels of neutralizing antibodies. Therefore, this finding underscores antigenic differences in strain 07-435 that are capable of inducing a neutralizing humoral response in the host [24,25].

Although the complete genome of BoGHV4 has been successfully sequenced, the functions of most of its genes remain unknown. Currently, only six strains of BoGHV4, all belonging to Genotype 1, have been sequenced [7,15,27,28,29]. This study presents two nearly complete genomes of BoGHV4, Genotypes 2 and 3, derived from clinical cases of cattle abortion in Argentina. The aim of this study was to characterize their genetic composition and to identify specific regions involved in antigenicity and pathogenicity in the host.

## 2. Material and Methods

### 2.1. Viruses

The BoGHV4 strains identified as 07-435 and 10-154 were isolated from vaginal discharges of aborted cows in Argentina [18] and classified as Genotypes 3 and 2, respectively, based on restriction patterns and partial sequences of gB and TK genes [18,30]. An enriched fraction of virions, free from cellular interferences, was obtained as described by Lété [9]. MDBK cells were infected with BoGHV4 strains at an MOI of 0.5. To reduce cellular contaminants, supernatants were collected at 96 h post infection before complete cell lysis. After the removal of cell debris by low-speed centrifugation (1000× *g*, 10 min, 4 °C), virions in the infected cell supernatant were collected by ultracentrifugation (100,000× *g*, 2 h, 4 °C) through a 30% (*w*/*v*) sucrose cushion. The obtained pellet, containing the enriched virus, was resuspended in medium (MEM-E) and stored at −80 °C until use.

### 2.2. Genomic Sequencing

Viral DNA was extracted from the previously obtained virion-enriched fraction, using a commercial kit (DNeasy Blood & Tissue Kit; Qiagen, Germany), according to the manufacturer’s instructions. The DNA concentration was determined by spectrometry, and its quality was evaluated in a 2% agarose gel by electrophoresis.

DNA samples were submitted for complete genome sequencing using next-generation DNA sequencing (NGS) technology (Novogene Co., Ltd. laboratory, Beijing, China). DNA quantification was performed with a Qubit^®^ 3.0 fluorometer. Samples with a total amount greater than 500 ng were qualified for library construction. Subsequently, the genomic DNA was randomly cut into short fragments of approximately 350 bp. The fragments obtained were subjected to library construction using the NEBNext^®^ DNA Library Prep Kit (New England Biolabs^®^, Ipswich, MA, USA), following the manufacturer’s instructions, and were later repaired at the ends by placing a poly-A tail and ligated with the Illumina adapter. Fragments with adapters were amplified by PCR, selected for size, and purified. Libraries were pooled and sequenced on Illumina platforms, according to their effective concentration and the amount of data required. Once the sequencing data were obtained, bioinformatic analysis (detection and annotation of SNPs, InDel, SV, and CNV according to the mapping results) and data filtration were carried out. Original sequencing data acquired by Illumina NovaSeq platform (Illumina, Inc. San Diego, CA, USA), recorded to image files, were first transformed into sequence reads using CASAVA software v1.82.2 (Illumina, Hayward, CA, USA). The sequences and the corresponding sequencing quality information were stored in a FASTQ file.

The ORFs of BoGHV4 isolates 07-435 and 10-154 were inferred and annotated using Geneious Prime 2023.0.4 software (https://www.geneious.com, accessed 26 January 2023) based on the genomic sequence of BoGHV4 strain 66-p-377 (GenBank accession number AF318573).

### 2.3. Genome Analyses

The obtained genome sequences were aligned and compared with the BoGHV4 complete genomes (CG) (Table S1) available in the NCBI database through the BLAST tool (https://blast.ncbi.nlm.nih.gov/Blast.cgi, accessed 6 July 2023). A maximum likelihood (ML) phylogenetic tree was constructed as described below.

Pairwise distances (p-distances) among the nucleotide (nt) sequences obtained in this study and those available in GenBank were calculated using MEGA v11.0.11 [31].

To explore the relationship between Argentinean BoGHV4 strains and various herpesvirus species, ML phylogenetic trees were constructed using three nucleotides sequence datasets: (i) DPOL from representative strains of *Alpha-*, *Beta-*, and *Gammaherpesvirinae* subfamilies; (ii) concatenated sequences of six highly conserved genes (gB, DPOL, major capsid protein, DNA packaging terminase 1, helicase-primase helicase subunit, and uracil-DNA glycosylase) from representative strains of the *Gammaherpesvirinae* subfamily; and (iii) partial TK gene sequences from representative BoGHV4 strains.

Maximum likelihood phylogenetic trees were constructed using the IQ-TREE web server (http://iqtree.cibiv.univie.ac.at/, accessed on 10 April 2024 [32]). The best-fitting nucleotide substitution model was determined with ModelFinder using the Bayesian Information Criterion [33]. The selected models were K3Pu + F + I, TVM + F + I + G4, SYM + I + G4, and JC for CG, DPOL, concatenated genes, and TK gen datasets, respectively. To assess the tree topology robustness, an ultrafast bootstrap (UFBoot) with 1000 bootstrap samples was performed. The resulting trees were annotated and visualized with Figtree v1.4.4 software [34].

### 2.4. ORF Analyses

#### 2.4.1. Sequence Analyses

Previous research conducted by our group revealed marked differences in molecular and biological features between 07-435 and 10-154 strains, including in vitro and in vivo replication, gene expression, antibody neutralization, and protein profile [23,24,25,35,36]. These findings prompted further characterization of the ORFs encoding IE2, gH, gL, gM, gB, gp180, and Bo17 genes. Additionally, three more ORFs were included for analyses: TK, typically used for diagnostic and genotyping purposes [18,30,37,38]; DPOL, used in phylogeny classification of *Alpha-*, *Beta-*, and *Gammaherpesvirinae* subfamilies [27,39]; and MCP due to its conserved nucleotide sequence [35,40].

The nt and deduced amino acid (aa) sequences of each ORF were aligned and compared using the MUSCLE algorithm in AliView v1.26 software [41,42]. Pairwise distances were calculated using MEGA v11.0.11.

Synonymous and non-synonymous substitutions per site and aa variation of Argentinean isolates were determined in comparison to the strain 66-p-347 (GenBank acc. num. AF318573) as a reference BoGHV4 Genotype 1.

Earlier observations revealed that isolate 07-435 induced high levels of neutralizing antibodies, contrasting with isolate 10-154, which failed to induce antibodies in ruminant species [23,25]. Machiels et al. [43] suggested that gp180 glycans provide shield to vulnerable antigenic epitopes against neutralizing antibodies. Thus, to explore 07-435 and 10-154 gp180 potential glycosylation, N- and O-glycosylated sites were predicted using the NetNglyc1.0 and NetOGlyc4.0 servers [44,45].

#### 2.4.2. Phylogenetic Comparisons

To assess the reliability of the analyzed ORFs for phylogenetic studies, the phylogenetic trees of each ORF were compared to the CG tree. The ML trees were constructed in the IQ-TREE web server as described above.

Tree comparison was performed by calculating the geodesic distance, which incorporates edge lengths and the tree topology [46]. Calculations were carried out using the TreeCmp software v2.0 (https://eti.pg.edu.pl/TreeCmp/, (accessed on 8 September 2023 [47]). The ORFs with the shortest geodesic distance were considered as the most similar to the CG tree. The pairs of compared trees were displayed using the Phylo.io application [48] in the TreeCmp software.

#### 2.4.3. Recombination Analyses

Significant tree topology differences may reflect BoGHV4 inter-strain recombination [49]. Therefore, evidence of recombination in the analyzed ORFs was evaluated using the methods implemented in the RDP v4.100 software [50] with the default settings. Only recombination events with *p* ≤ 0.05 detected by at least four methods were considered.

## 3. Results

### 3.1. Genomic Sequencing

Sequence assembling resulted in a LUR of 108,381 nt in length for strain 07-435 and 108,667 nt for strain 10-154. Both LURs encoded 79 ORFs (Figure S1). The obtained genomes were deposited in the GenBank database under the accession numbers OQ709765 and OQ709766.

### 3.2. Genome Analyses

Phylogenetic analysis of CG showed three main clades. Strain 07-435 (Genotype 3) formed a distinct clade separate from the remaining genomes. Strain 10-154 (Genotype 2) clustered within a subclade alongside Genotype 1 strains, sharing a common ancestor with USA strains 66-p-347 and SD16-38. The third main clade included two Genotype 1 isolates, FMV09-1180503 and SD16-49 (Figure S2).

The nt p-distance between strains 07-435 and 10-154 was 3.34%. In comparison to BoGHV4 Genotype 1, strain 07-435 showed a p-distance of 3.01% to 3.33%, whereas strain 10-154 exhibited values between 1.29% and 1.43%. The genomic sequences of the six BoGHV4 Genotype 1 isolates displayed high similarity, with differences ranging from 0.03 to 1.14%. This level of divergence was lower than that observed in the Argentinean isolates (Table 1).

**Figure 1:**
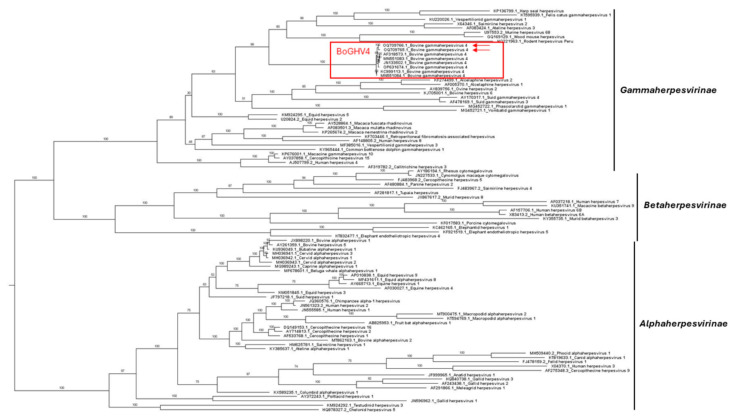
Maximum likelihood phylogenetic trees based on DNA polymerase (DPOL) gene of *Alpha-*, *Beta*-, and *Gammaherpesvirinae* subfamilies. Ultrafast bootstrap values (1000 replicates) are shown. The tree is midpoint-rooted for clarity. Bovine gammaherpesvirus 4 (BoGHV4) is highlighted by a red box, and BoGHV4 strains reported in the current study are highlighted by red arrows.

**Figure 2:**
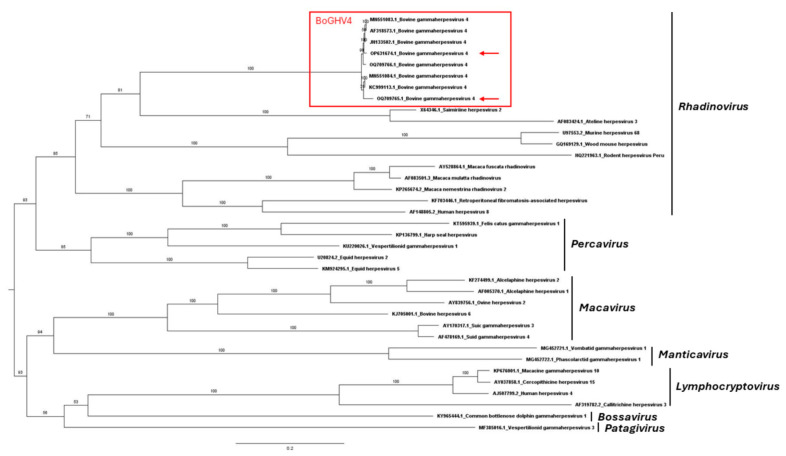
Maximum likelihood phylogenetic trees based on six concatenated genes (glycoprotein B, DNA polymerase, major capsid protein, DNA packaging terminase 1, helicase-primase helicase subunit, and uracil-DNA glycosylase) of the *Gammaherpesvirinae* subfamily. Ultrafast bootstrap values (1000 replicates) are shown. The tree is midpoint-rooted for clarity. Bovine gammaherpesvirus 4 (BoGHV4) is highlighted by a red box, and BoGHV4 strains reported in the current study are highlighted by red arrows.

The DPOL phylogenetic tree (Figure 1) positioned the two Argentinean strains within the *Gammaherpesvirinae* subfamily with other BoGHV4 strains. Similarly, the phylogeny of the six concatenated sequences of the *Gammaherpesvirinae* subfamily showed the Argentinean strains within the *Rhadinovirus* genus (Figure 2). Both strains from Argentina shared a highly supported cluster (UFBoot = 100%) with BoHGV4 sequences.

**Figure 3:**
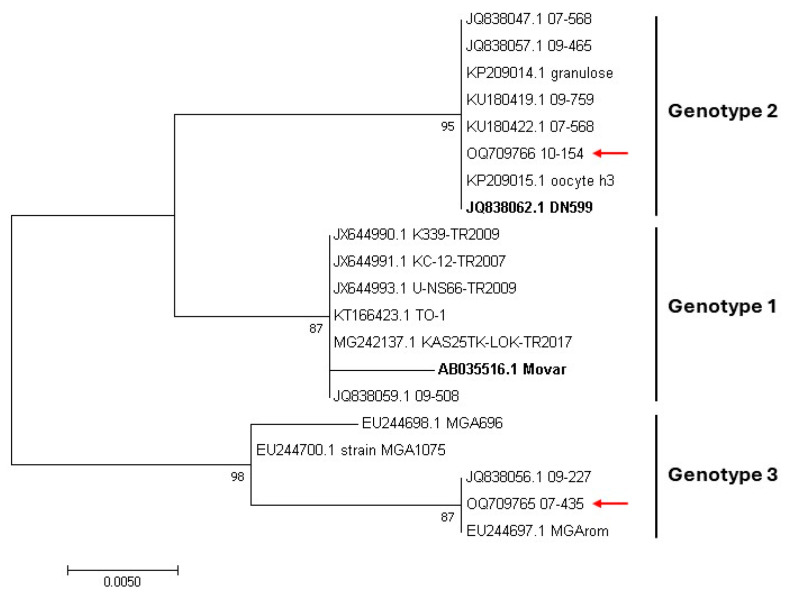
Maximum likelihood phylogenetic trees based on partial thymidine kinase (TK) gene of bovine gammaherpesvirus 4 (BoGHV4) Genotypes 1, 2, and 3. Ultrafast bootstrap values (1000 replicates) are shown. The tree is midpoint-rooted for clarity. BoGHV4 strains reported in the current study are highlighted by red arrows. The reference strains Movar and DN599 are in bold.

The phylogenetic analysis based on the partial TK region revealed three well-supported clades representing BoGHV4 Genotypes 1, 2, and 3 (Figure 3). Strain 07-435 clustered with isolates previously classified as Genotype 3, whereas strain 10-154 grouped with isolates identified as Genotype 2.

### 3.3. ORF Analyses

#### 3.3.1. Sequence Analyses

Nucleotide and aa p-distances varied among the strains depending on the analyzed ORF (Tables S2–S11). Briefly, higher values were observed between Argentinean strain 07-435 and Genotype 1 isolates, except for gB and Bo17 ORFs (Tables S3 and S10). Conversely, the Argentinean strain 10-154 exhibited greater nt and aa p-distances only in the gL, gp180, and IE2 ORFs compared to Genotype 1 strains (Tables S6–S8).

Detailed analyses of Argentinean strains were conducted using the 66-p-347 (AF318573) genome as a representative BoGHV4 Genotype 1. Pairwise comparisons showed higher aa p-distance values compared to nt p-distance in the gB, gH, gL, gp180, IE2, and Bo17 for 07-435 strain (Tables S3, S4, S6–S8 and S10). This suggests that most substitutions occurring in those ORFs were of non-synonymous type. Conversely, the strain 10-154 exhibited fewer ORFs with greater aa diversity, namely gH, gL, and gp180 (Tables S4, S5 and S7). The remaining analyzed ORFs showed similar or lower aa p-distance compared to nt values.

**Figure 4:**
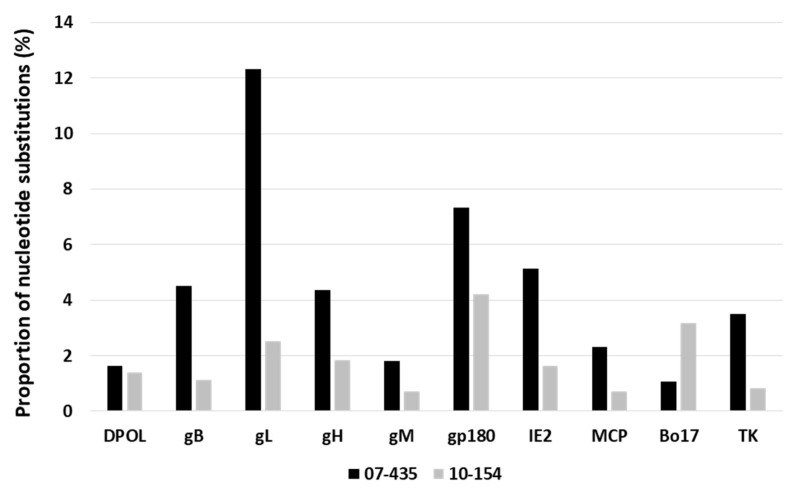
Proportions of nucleotide substitutions in bovine gammaherpesvirus 4 strains 07-135 and 10-154 using the genome 66-p-347 (AF318573) as reference.

Due to the findings described above, the nt substitutions and resulting aa changes between the Argentinean strains and isolate 66-p-347 were further explored by sequence alignment. Overall, the analyzed ORFs showed more diversity in strain 07-435 than in 10-154, with the exception of the Bo17 sequence. The ORFs gL, gp180, and IE2 presented the highest proportions of nt substitutions, whereas DPOL, gM, and MCP displayed the lowest levels of nucleotide substitutions (Figure 4).

**Figure 5:**
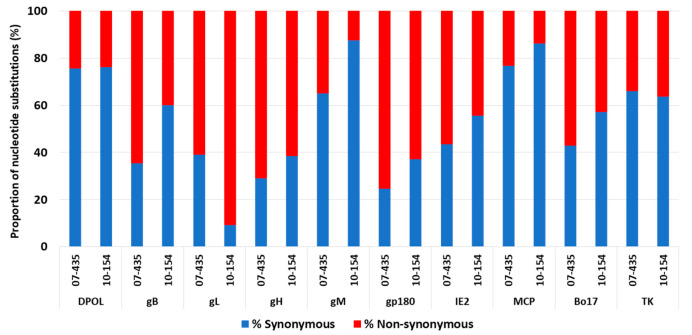
Proportions of synonymous and non-synonymous nucleotide substitutions in bovine gammaherpesvirus 4 strains 07-135 and 10-154 using the genome 66-p-347 (AF318573) as reference.

Further examination showed that the proportions of synonymous and non-synonymous substitutions in the Argentinean isolates differed from ORF-to-ORF sequences compared to strain 66-p-347 (Figure 4). The number of non-synonymous substitutions was higher than the synonymous ones in gB, gL, gH, gp180, IE2, and Bo17 for isolate 07-435; thus, more aa changes were observed. On the contrary, isolate 10-154 exhibited more non-synonymous than synonymous substitutions in gL, gH, and gp180 (Figure 5).

The NetNGlyc 1.0 and NetOGlyc 4.0 servers identified 131 potential N-glycosylated sites and 7 potential O-glycosylated sites in 07-435 gp180, whereas 126 N-glycosylated sites and 5 O-glycosylated sites were predicted for 10-154 gp180. These values are similar to those reported for the gp180 of the V.test strain [43].

#### 3.3.2. Phylogenetic Comparisons

The shortest geodesic distances between CG phylogeny and the ORFs were observed for TK, showing the most similar topology compared to that displayed for CG. In contrast, gB showed the largest geodesic distances (Table S12). However, all the ORF trees differed from the CG tree and resulted in distinct phylogenetic topologies (Figures S3–S12).

#### 3.3.3. Recombination Analyses

Only the Argentinean strains 07-435 and 10-154 were identified as possible recombinants. The recombination events were observed in DPOL (n = 2), gB (n = 2), and gH (n = 1). However, four out of the five signals were indicated as possibly false events. No evidence of recombination was detected in the rest of the analyzed ORFs (Table S13).

## 4. Discussion

Emerging and re-emerging viral agents constitute an object of attention within the development of the bovine industry worldwide. With this background, it is necessary to maintain a virus surveillance system based on isolation and molecular characterization. BoGHV4 emerged in Argentina as a pathogenic agent in bovine production systems just less than two decades ago [18]. Considering the biological properties diversity of the BoGHV4 variants [30,35], in this work, whole genome sequence-based analysis of two Argentinean strains of BoGHV4 was carried out to understand their differential characteristics with respect to the prototypes described until now.

The two genome sequences reported in this study add significantly to the six BoGHV4 genomes previously described [7,15,27,28,29]. Both Argentinean isolates presented the highest nt p-distance values, indicating a greater level of divergence.

Phylogenetic and sequence analyses of CG placed the strain 07-435 in a distant clade with the highest nt p-distance, showing a greater level of divergence with the rest of the strains. These results confirm earlier studies that proposed isolate 07-435 as a different genotype designated as Genotype 3 based on gB and TK partial sequences [18,35].

The Argentinean strain 10-154 exhibited intermediate p-distance values when compared to those observed between the Genotype 1 strains and isolate 07-435. This level of divergence supports earlier studies that classified isolate 10-154 as Genotype 2 [35]. Interestingly, despite its classification as Genotype 2, isolate 10-154 clustered with two BoGHV4 Genotype 1 strains from USA, suggesting a potential origin from Genotype 1 strains.

As expected, the DPOL and concatenated sequences phylogenetic analyses confirmed that the isolates studied herein, along with previously described BoGHV4 genomes, belong to the *Gammaherpesvirinae* subfamily, *Rhadinovirus* genus [1,51,52,53]. In both trees, Argentinean isolates shared a cluster with BoGHV4 Genotype 1 strains for which the CG is available, but the Argentinean sequences did not clearly grouped within Genotype 1, suggesting that both strains belong to a different Genotype. Consequently, a further phylogenetic tree was constructed based on the partial TK gene that is widely used for BoGHV4 classification, which included reference and previously studied strains. The data suggest the Argentinean strains to be Genotype 2 and 3, as proposed earlier by Verna et al. [18] and Pérez et al. [30].

The high genomic divergence between the Argentinean and the available BoGHV4 CG as well as the body of research showing differences in biological features between isolates 07-435 and 10-154 [23,24,25,35,36] motivated the detailed analysis of different ORFs. In accordance with the CG divergency of both isolates, variations in nt and aa were also observed in the 10 further explored ORFs.

Regarding strain 07-435, notable variability was observed in the envelope glycoproteins gB, gL, gH, and gp180. Moreover, these ORFs presented higher non-synonymous than synonymous substitutions, leading to several aminoacidic changes. This finding was unexpected since the gB, gL, and gH glycoproteins, the core of the machinery entry for herpesvirus, are highly conserved [54,55]. Substantial gp180 divergence between BoGHV4 strains was previously reported by Michaels et al. [10], who argued that this protein might not require a highly specific aa sequence for its function.

The humoral response in cattle infected with BoGHV4 is characterized by low or absence of neutralizing antibody levels [56,57,58]. A mechanism involved in the limitation or blocking of virion neutralization by antibodies seems to be the N- and O-glycans of the BoGHV4 gp180, which masks antigenic epitopes in gB, gH, and gL [10]. Our research group showed that isolate 07-435 induced the production of high levels of neutralizing antibodies in different ruminant species, including cattle, deer, sheep, and goat [23,24,25]. On the contrary, in the same studies, the absence of neutralizing antibody response was observed for isolate 10-154.

Machiels et al. [10] showed that gp180-deficient virions were sensitive to neutralization by anti-BoGHV4 serum. Based on those findings, in the present study, potential O- and N-glycosylation sites were evaluated for both 07-435 and 10-154 isolates. Both strains showed high potential O-glycosylation and no clear difference in N-glycosylation. Therefore, it is not possible to propose a modification in gp180 glycosylation in strain 07-435 as the cause of neutralization susceptibility. Further in vitro and in vivo studies are needed to deepen into the mechanisms involved in BoGHV4 susceptibility to neutralizing antibodies.

Markine-Goriaynoff et al. [13] studied the non-synonymous to synonymous substitutions ratio in the Bo17 gene across nine representative strains of BoGHV4. The authors demonstrated a pronounced restriction on non-synonymous substitutions. In the present study, strain 10-154 showed greater diversification than 07-435 for Bo17, which could affect the post-translational modifications of structural protein of the virus. These modifications, when occurring in vivo, have the potential to impact the tropism of the virus as well as its sensitivity to antibody and complement neutralization [13].

Nucleotides sequences of DPOL and MCP, involved in DNA synthesis and virion structural component, showed low nucleotide substitutions with a high proportion of synonymous substitutions for the Argentinean strains. This demonstrates the high conservation of both genes in BoGHV4 and across herpesvirus species [59,60].

Considering the crucial role that the IE2 gene plays in the initiation and facilitation of the progression of BoGHV-4 lytic replication [4,61], the higher nucleotide substitution rate and increased non-synonymous substitution observed in strain 07-435 could explain the difference in the permissibility and replication kinetics of the Argentinean strains under study, as previously reported [24].

The evolutionary history of a gene might be different from that of the CG; therefore, phylogenetic trees and virus classification relying on individual genes must be treated with caution [62]. Several studies have shown different BoGHV4 tree topologies depending on the genetic region used [30,38,49,63]. In the present study, to a better understand the genomic classification of BoGHV4, phylogenetic trees between the CG and the explored ORFs were performed.

Partial nt sequences of gB and TK have been widely used for BoGHV4 classification and diagnostic purposes [17,18,21,29,30,37,63]. The phylogenetic comparison showed that TK sequences displayed the most similar tree and the shortest geodesic distance compared to the CG. This supports TK as a suitable choice for BoGHV4 phylogenetic and classification analyses. On the contrary, gB showed the poorest agreement with the CG phylogenetic tree, which might imply that the analysis based only on gB sequences is not reliable enough. In this sense, Verna et al. [18] already suggested that more than one gene should be used to create an accurate picture of BoGHV4 genomic diversity.

Herpesvirus diversification and evolution have been partly attributed to the recombination process [6,64]. Recombination events have been demonstrated for different herpesvirus species [65,66,67,68], including BoGHV4 [49]. In this study, recombination signals were detected only in the Argentinean strains, although four out of the five signals were identified as possibly false recombination events. It appears that recombination between divergent BoGHV4 strains has been relatively infrequent [49]. Therefore, the hypothesis of recombination as a cause of significant topological differences among trees [49,69] was not supported in the performed analyses.

## 5. Conclusions

To the best of the authors’ knowledge, this is the first report of the near CG of Argentinean BoGHV4 strains and the first Genotype 2 and 3 genomes described in the literature. The inclusion of these sequences stands as a noteworthy expansion to the currently limited pool of BoGHV4 genomes while providing an important basis to increase the knowledge of local isolates.

It is important to note that these results were obtained considering only the six BoGHV4 genomes available in the GenBank database and the two genomes reported herein. Therefore, future analyses including more genomes will consolidate our understanding of the genetic diversity of BoGHV4, which is important for developing diagnostic methods and prevention.

The combination of PCR amplification and next-generation sequencing (NGS) analysis of viral strains isolated from clinical cases of BoGHV4 could be a powerful approach to investigate samples that would otherwise be difficult or impossible to study.

## Figures and Tables

**Table 1:** Pairwise nucleotide distances (%) among BoGHV-4 genomes available in the GenBank database. Argentinean genomes sequenced in the present study are shown in bold.

	66-p-347	SD16-38	HB-ZJK	FMV09	SD16-49	V.test	10-154	07-435
66-p-347	-							
SD16-38	0.03	-						
HB-ZJK	1.03	1.03	-					
FMV09	1.14	1.14	1.07	-				
SD16-49	1.13	1.12	1.04	0.06	-			
V.test	0.82	0.82	0.87	1.05	1.04	-		
**10-154**	1.32	1.33	1.43	1.39	1.39	1.29	-	
**07-435**	3.27	3.29	3.21	3.02	3.01	3.33	**3.34**	-

## Data Availability

The nucleotide sequences were deposited in GenBank under the accession numbers OQ709765 and OQ709766.

## Supplementary Materials

The following supporting information can be downloaded at: https://www.mdpi.com/article/10.3390/v16050739/s1. Table S1: Bovine Gammaherpesvirus 4 genome sequences available in the GenBank database; Table S2: Pairwise nucleotide and amino acid distances of the DPOL gene (ORF 9) among the Bovine Gammaherpesvirus 4 strains available in the GenBank database; Table S3: Pairwise nucleotide and amino acid distances of the gB gene (ORF 8) among the Bovine Gammaherpesvirus 4 strains available in the GenBank database; Table S4: Pairwise nucleotide and amino acid distances of the gH gene (ORF 22) among the Bovine Gammaherpesvirus 4 strains available in the GenBank database; Table S5: Pairwise nucleotide and amino acid distances of the gM gene (ORF 39) among the Bovine Gammaherpesvirus 4 strains available in the GenBank database; Table S6: Pairwise nucleotide and amino acid distances of the gL gene (ORF 47) among the Bovine Gammaherpesvirus 4 strains available in the GenBank database; Table S7: Pairwise nucleotide and amino acid distances of the gp80 gene (ORF Bo10) among the Bovine gammaherpesvirus 4 strains available in the GenBank database; Table S8: Pairwise nucleotide and amino acid distances of the IE2 gene (ORF 50) among the Bovine Gammaherpesvirus 4 strains available in the GenBank database; Table S9: Pairwise nucleotide and amino acid distances of the MCP gene (ORF 25) among the Bovine Gammaherpesvirus 4 strains available in the GenBank database; Table S10: Pairwise nucleotide and amino acid distances of the Bo17 among the Bovine Gammaherpesvirus 4 strains available in the GenBank database; Table S11: Pairwise nucleotide and amino acid distances of the TK gene (ORF 21) among the Bovine Gammaherpesvirus 4 strains available in the GenBank database; Table S12: Geodesic distance between the Bovine Gammaherpesvirus complete genome and ORF phylogenetic trees; Table S13: Possible recombination events in the analyzed Bovine Gammaherpesvirus 4 ORFs; Figure S1: Organization of BoGHV-4 07-435 and 10-154 genome map; Figure S2: Maximum likelihood phylogenetic trees based on Bovine Gammaherpesvirus 4 genomes; Figure S3: Comparison of phylogenetic trees obtained the Bovine Gammaherpesvirus 4 genome and DPOL sequences; Figure S4: Comparison of phylogenetic trees obtained the Bovine Gammaherpesvirus 4 genome and gB sequences; Figure S5: Comparison of phylogenetic trees obtained the Bovine Gammaherpesvirus 4 genome and gH sequences; Figure S6: Comparison of phylogenetic trees obtained the Bovine Gammaherpesvirus 4 genome and gL sequences; Figure S7: Comparison of phylogenetic trees obtained the bovine herpesvirus 4 genome and gM sequences; Figure S8: Comparison of phylogenetic trees obtained the Bovine Gammaherpesvirus 4 genome and gp180 sequences; Figure S9: Comparison of phylogenetic trees obtained the Bovine Gammaherpesvirus 4 genome and IE2 sequences; Figure S10: Comparison of phylogenetic trees obtained the Bovine Gammaherpesvirus 4 genome and MCP sequences; Figure S11: Comparison of phylogenetic trees obtained the Bovine Gammaherpesvirus 4 genome and Bo17 sequences; Figure S12: Comparison of phylogenetic trees obtained the Bovine Gammaherpesvirus 4 genome and TK sequences.
