# Supplementary material for: Whole Genome Sequence-Based Analysis of Bovine Gammaherpesvirus 4 Isolated from Bovine Abortions
Source: Viruses. 2024 May 8;16(5):739. doi: 10.3390/v16050739 (PMC11125609; doi:10.3390/v16050739)
Supplement: Supplementary file 1 [file viruses-16-00739-s001.zip › viruses-2959051-supplementary.pdf]

Table S1. Bovine Gammaherpesvirus 4 genome sequences available in the GenBank database.

Strain	Accession number	Country	Year	Length, nt	Genotype	Reference
V.test	JN133502	Belgium	1981	108241	1	Palmeira et al., 2011
HB-ZJK	OP631674	China	2022	108778	1	Guo et al., 2023
SD16-38	MN551083	USA	2016	108668	1	Bauermann et al., 2022
66-p-347	AF318573	USA	-	108873	1	Zimmermann et al., 2001
FMV09	KC999113	Canada	2009	108349	1	Cagnon et al., 2017
SD16-49	MN551084	USA	2016	108477	1	Bauermann et al., 2022

Table S2. Pairwise nucleotide and amino acid distances of the DPOL gene (ORF 9) among the Bovine Gammaherpesvirus 4 strains available in the GenBank database. Argentinean strains are shown in bold.

	66-p-347	SD16-38	HB-ZJK	FMV09	SD16-49	V.test	**10-154**	**07-435**
					Amino acid distance, %			
66-p-347	-	0.1	0.2	0.8	0.8	0.1	0.9	1.19
SD16-38	0.03	-	0.1	0.7	0.7	0	0.8	1.09
HB-ZJK	0.7	0.66	-	0.6	0.6	0.1	0.7	1
FMV09	1.19	1.16	1.13	-	0	0.7	1.09	1.39
SD16-49	1.19	1.16	1.13	0	-	0.7	1.09	1.39
V.test	0.17	0.13	0.66	1.16	1.16	-	0.8	1.09
**10-154**	1.39	1.36	1.33	0.99	0.99	1.29	-	0.7
**07-435**	1.62	1.59	1.56	1.33	1.33	1.52	0.76	-
			Nucleotide distance, %					

Table S3. Pairwise nucleotide and amino acid distances of the gB gene (ORF 8) among the Bovine Gammaherpesvirus 4 strains available in the GenBank database. Argentinean strains are shown in bold.

	66-p-347	SD16-38	HB-ZJK	FMV09	SD16-49	V.test	**10-154**	**07-435**
					Amino acid distance, %			
66-p-347	-	0	0.34	7.32	7.32	0.23	1.14	8.08
SD16-38	0	-	0.34	7.32	7.32	0.23	1.14	8.08
HB-ZJK	0.27	0.27	-	7.21	7.21	0.34	1.03	7.97
FMV09	4.15	4.15	4.19	-	0	7.32	7.21	0.92
SD16-49	4.15	4.15	4.19	0	-	7.32	7.21	0.92
V.test	0.08	0.08	0.27	4.15	4.15	-	1.14	8.08
**10-154**	1.14	1.14	1.18	4.11	4.11	1.14	-	**7.97**
**07-435**	4.58	4.58	4.61	1.04	1.04	4.58	**4.84**	-
		Nucleotide distance, %						

Table S4. Pairwise nucleotide and amino acid distances of the gH gene (ORF 22) among the Bovine Gammaherpesvirus 4 strains available in the GenBank database. Argentinean strains are shown in bold.

	66-p-347	SD16-38	HB-ZJK	FMV09	SD16-49	V.test	**10-154**	**07-435**
					Amino acid distance, %			
66-p-347	-	0	1.56	5.52	5.52	0.85	3.11	8.84
SD16-38	0	-	1.56	5.52	5.52	0.85	3.11	8.84
HB-ZJK	0.56	0.56	-	6.22	6.22	0.99	3.82	9.56
FMV09	2.87	2.87	3.06	-	0	5.66	3.39	8.56
SD16-49	2.87	2.87	3.06	0	-	5.66	3.39	8.56
V.test	0.28	0.28	0.38	2.97	2.97	-	3.11	8.84
**10-154**	1.84	1.84	2.02	1.69	1.69	1.84	-	**9.7**
**07-435**	4.42	4.42	4.65	4.61	4.61	4.42	**5.08**	-
		Nucleotide distance, %						

Table S5. Pairwise nucleotide and amino acid distances of the gM gene (ORF 39) among the Bovine Gammaherpesvirus 4 strains available in the GenBank database. Argentinean strains are shown in bold.

	66-p-347	SD16-38	HB-ZJK	FMV09	SD16-49	V.test	**10-154**	**07-435**
					Amino acid distance, %			
66-p-347	-	0	0.27	0.27	0.27	0.27	0.27	1.9
SD16-38	0	-	0.27	0.27	0.27	0.27	0.27	1.9
HB-ZJK	0.54	0.54	-	0	0	0.54	0.54	1.63
FMV09	0.45	0.45	0.09	-	0	0.54	0.54	1.63
SD16-49	0.45	0.45	0.09	0	-	0.54	0.54	1.63
V.test	0.72	0.72	0.9	0.81	0.81	-	0.54	2.17
**10-154**	0.72	0.72	0.9	0.81	0.81	0.81	-	**2.17**
**07-435**	1.8	1.8	1.62	1.53	1.53	1.98	**1.98**	-
		Nucleotide distance, %						

Table S6. Pairwise nucleotide and amino acid distances of the gL gene (ORF 47) among the Bovine Gammaherpesvirus 4 strains available in the GenBank database. Argentinean strains are shown in bold.

	66-p-347	SD16-38	HB-ZJK	FMV09	SD16-49	V.test	**10-154**	**07-435**
					Amino acid distance, %			
66-p-347	-	2.1	2.8	2.8	3.5	2.86	6.29	18.31
SD16-38	0.69	-	2.1	2.1	2.8	2.14	6.29	18.31
HB-ZJK	0.93	0.69	-	1.4	2.1	1.43	5.59	19.01
FMV09	1.16	0.93	0.69	-	0.7	0.71	4.9	18.31
SD16-49	1.62	1.39	1.16	0.46	-	1.43	5.59	18.31
V.test	1.18	0.95	0.71	0.47	0.95	-	5	18.57
**10-154**	2.55	2.78	2.55	2.08	2.55	2.36	-	**19.72**
**07-435**	12.59	12.35	12.35	12.35	12.82	12.29	**13.29**	-
		Nucleotide distance, %						

Table S7. Pairwise nucleotide and amino acid distances of the gp80 gene (ORF Bo10) among the Bovine Gammaherpesvirus 4 strains available in the GenBank database. Argentinean strains are shown in bold.

	66-p-347	SD16-38	HB-ZJK	FMV09	SD16-49	V.test	**10-154**	**07-435**
					Amino acid distance, %			
66-p-347	-	0	0.37	0.37	0.73	0.37	8.06	16.18
SD16-38	0	-	0.37	0.37	0.73	0.37	8.06	16.18
HB-ZJK	0.12	0.12	-	0	0.37	0	7.69	15.81
FMV09	0.12	0.12	0	-	0.37	0	7.69	15.81
SD16-49	0.24	0.24	0.12	0.12	-	0.37	8.06	16.18
V.test	0.36	0.36	0.24	0.24	0.36	-	7.69	15.81
**10-154**	4.26	4.26	4.14	4.14	4.26	4.14	-	**10.55**
**07-435**	7.45	7.45	7.33	7.33	7.45	7.33	**5.07**	-
			Nucleotide distance, %					

Table S8. Pairwise nucleotide and amino acid distances of the IE2 gene (ORF 50) among the Bovine Gammaherpesvirus 4 strains available in the GenBank database. Argentinean strains are shown in bold.

	66-p-347	SD16-38	HB-ZJK	FMV09	SD16-49	V.test	**10-154**	**07-435**
					Amino acid distance, %			
66-p-347	-	0	0.36	0	0	0.54	1.5	8.15
SD16-38	0	-	0.36	0	0	0.54	1.5	8.15
HB-ZJK	0.24	0.24	-	0.36	0.36	0.91	1.87	8.15
FMV09	0.24	0.24	0.36	-	0	0.54	1.5	8.15
SD16-49	0.24	0.24	0.36	0	-	0.54	1.5	8.15
V.test	0.3	0.3	0.42	0.42	0.42	-	1.5	8.15
**10-154**	1.68	1.68	1.8	1.8	1.8	1.62	-	**7.66**
**07-435**	5.12	5.12	5.18	5.18	5.18	5.06	**4.91**	-
			Nucleotide distance, %					

Table S9. Pairwise nucleotide and amino acid distances of the MCP gene (ORF 25) among the Bovine Gammaherpesvirus 4 strains available in the GenBank database. Argentinean strains are shown in bold.

	66-p-347	SD16-38	HB-ZJK	FMV09	SD16-49	V.test	**10-154**	**07-435**
					Amino acid distance, %			
66-p-347	-	0	0.58	0.36	0.36	0.22	0.29	1.46
SD16-38	0	-	0.58	0.36	0.36	0.22	0.29	1.46
HB-ZJK	1.19	1.19	-	0.51	0.51	0.36	0.44	1.24
FMV09	0.8	0.8	1.33	-	0	0.15	0.22	1.38
SD16-49	0.78	0.78	1.31	0.02	-	0.15	0.22	1.38
V.test	0.78	0.78	1.16	0.32	0.29	-	0.07	1.24
**10-154**	0.7	0.7	1.24	0.34	0.32	0.27	-	**1.31**
**07-435**	2.3	2.3	1.26	2.5	2.47	2.43	**2.4**	-
		Nucleotide distance, %						

Table S10. Pairwise nucleotide and amino acid distances of the Bo17 among the Bovine Gammaherpesvirus 4 strains available in the GenBank database. Argentinean strains are shown in bold.

	66-p-347	SD16-38	HB-ZJK	FMV09	SD16-49	V.test	**10-154**	**07-435**
					Amino acid distance, %			
66-p-347	-	0	1.59	1.83	1.83	1.36	4.09	1.83
SD16-38	0	-	1.59	1.83	1.83	1.36	4.09	1.83
HB-ZJK	1.06	1.06	-	1.14	1.14	1.59	4.32	1.14
FMV09	1.21	1.21	0.61	-	0.46	1.83	4.34	0.46
SD16-49	1.14	1.14	0.53	0.23	-	1.83	4.34	0.46
V.test	0.98	0.98	1.13	1.29	1.21	-	4.55	1.83
**10-154**	3.17	3.17	3.02	3.19	3.11	3.55	-	**4.34**
**07-435**	1.06	1.06	0.45	0.3	0.23	1.14	**3.04**	-
		Nucleotide distance, %						

Table S11. Pairwise nucleotide and amino acid distances of the TK gene (ORF 21) among the Bovine Gammaherpesvirus 4 strains available in the GenBank database. Argentinean strains are shown in bold.

	66-p-347	SD16-38	HB-ZJK	FMV09	SD16-49	V.test	**10-154**	**07-435**
					Amino acid distance, %			
66-p-347	-	0	1.35	1.35	1.35	0.67	0.9	3.6
SD16-38	0	-	135	1.35	1.35	0.67	0.9	3.6
HB-ZJK	1.27	1.27	-	1.8	1.8	1.57	1.35	3.6
FMV09	1.12	1.12	1.79	-	0	1.57	1.35	3.82
SD16-49	1.12	1.12	1.79	0	-	1.57	1.35	3.82
V.test	0.37	0.37	1.35	1.05	1.05	-	1.12	3.82
**10-154**	0.82	0.82	0.75	1.35	1.35	0.9	-	**3.15**
**07-435**	3.51	3.51	3.06	3.66	3.66	3.59	**3.14**	-
		Nucleotide distance, %						

Table S12. Geodesic distance between the bovine Gammaherpesvirus complete genome and ORF phylogenetic trees.

**ORF**	**Geodesic distance***
TK	0.0145
IE2	0.015
MCP	0.0195
gM	0.0249
gH	0.0261
DPOL	0.033
gp80	0.0394
Bo17	0.0396
gB	0.0501
gL	0.0987

*ORFs are ordered from shortest to longest geodesic distance.

Table S13. Possible recombination events in the analyzed Bovine Gammaherpesvirus 4 ORFs.

Event recombination number	Sequence#	Major parent	Minor parent
**DPOL gene**			
1*	OQ709765_07-435	Unknown	MN551084_SD16-49
	OQ709766_10-154	Unknown	AF318573_66-p-347
2*	OQ709765_07-435	Unknown	MN551084_SD16-38
	OQ709766_10-154	Unknown	AF318573_66-p-347
**gB gene**			
1*	OQ709765_07-435*	Unknown	MN551084_SD16-49
2	OQ709766_10-154	OP631674_HB-ZJK	MN551084_SD16-49
**gH gene**			
1*	OQ709766_10-154*	OP631674_HB-ZJK	MN551084_SD16-49

#No recombination event was detected in gL, gM, gp80, IE2, MCP, Bo17, and TK.

*Recombination might not have caused this signal.

07-435

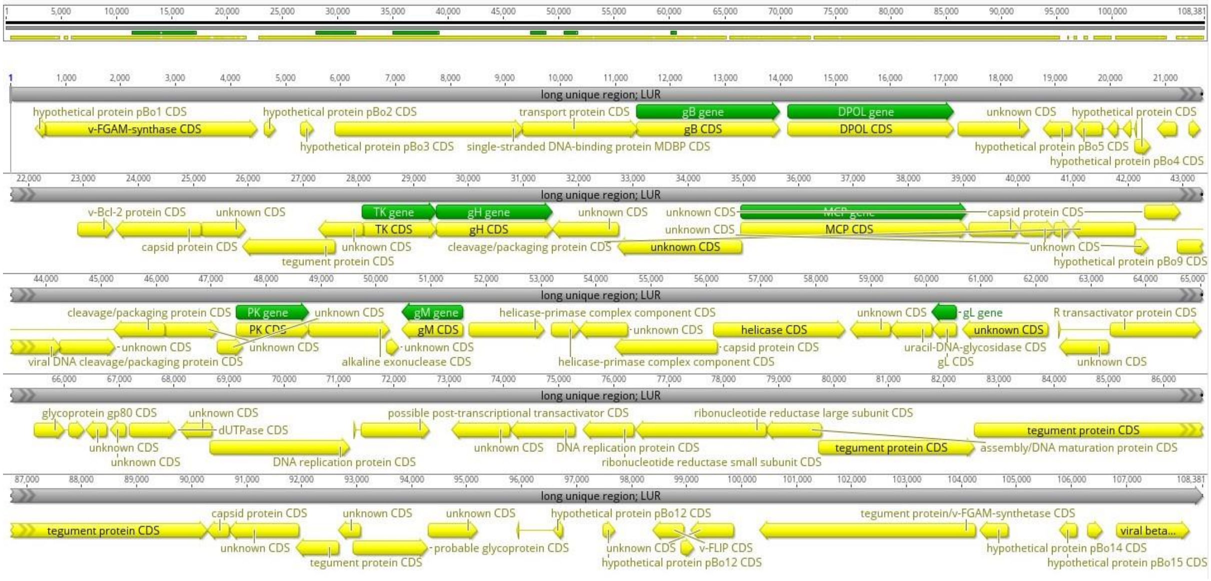

10-154

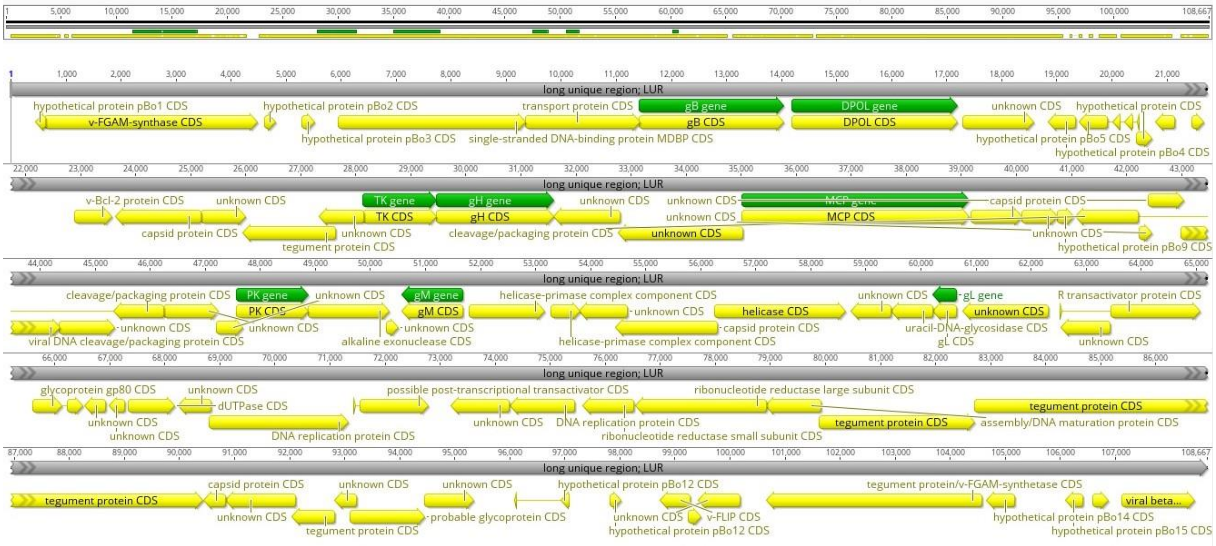

Figure S1. Organization of BoGHV-4 07-435 and 10-154 genome map. The genomes contain a long unique sequence region (LUR) and 79 open reading frames. The map was drawn by Geneious Prime 2023.0.4 software (https://www.geneious.com).

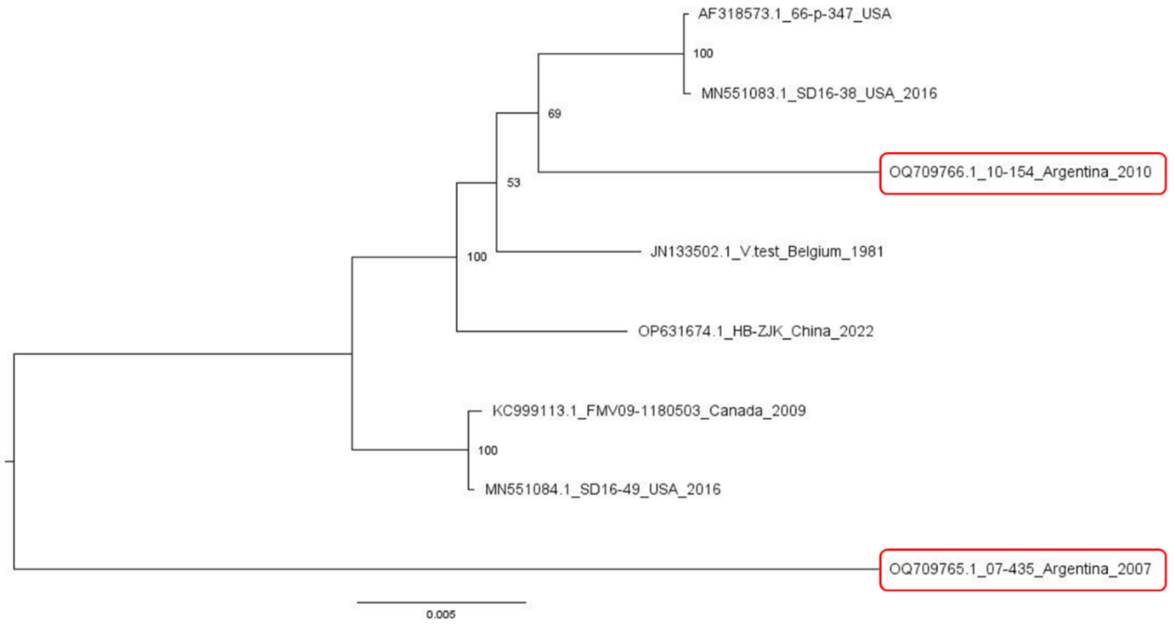

Figure S2. Maximum likelihood phylogenetic trees based on Bovine Gammaherpesvirus 4 genomes. Ultrafast bootstrap values (1000 replicates) are shown. The tree is midpoint-rooted for clarity. Bovine Gammaherpesvirus 4 genomes reported in the current study are highlighted by red boxes.

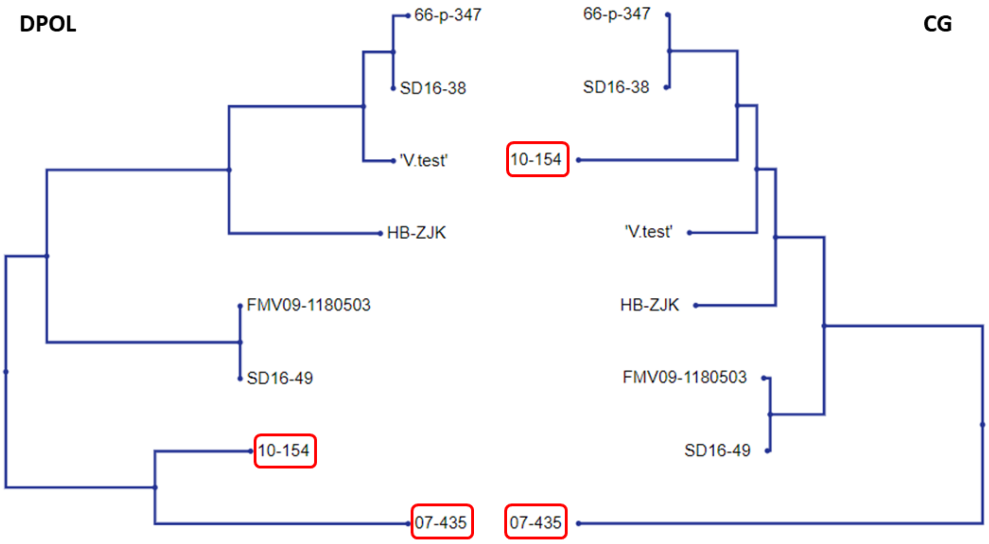

Figure S3. Comparison of phylogenetic trees obtained the Bovine Gammaherpesvirus 4 genome (right) and DPOL (left) sequences. Bovine herpesvirus 4 strains reported in the current study are highlighted by red boxes. The trees were displayed using the Phylo.io application (Robinson et al., 2016) in the TreeCmp software v2.0 (Goluch et al., 2020).

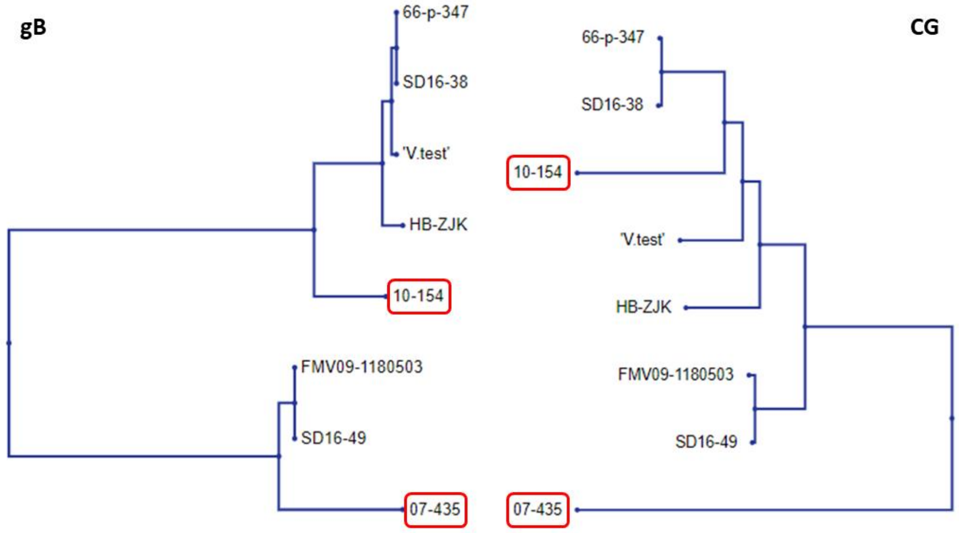

Figure S4. Comparison of phylogenetic trees obtained the Bovine Gammaherpesvirus 4 genome (right) and gB (left) sequences. Bovine herpesvirus 4 strains reported in the current study are highlighted by red boxes. The trees were displayed using the Phylo.io application (Robinson et al., 2016) in the TreeCmp software v2.0 (Goluch et al., 2020).

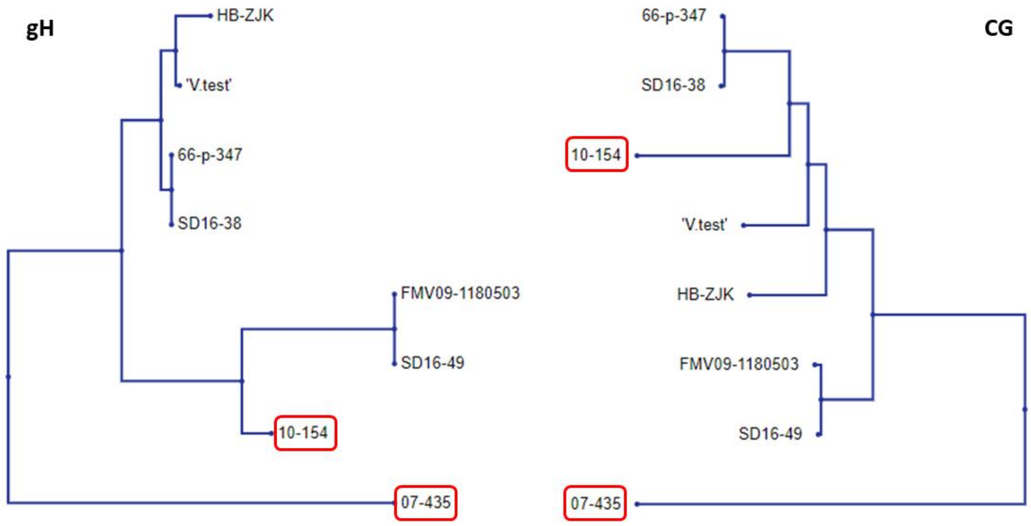

Figure S5. Comparison of phylogenetic trees obtained the Bovine Gammaherpesvirus 4 genome (right) and gH (left) sequences. Bovine Gammaherpesvirus 4 strains reported in the current study are highlighted by red boxes. The trees were displayed using the Phylo.io application (Robinson et al., 2016) in the TreeCmp software v2.0 (Goluch et al., 2020).

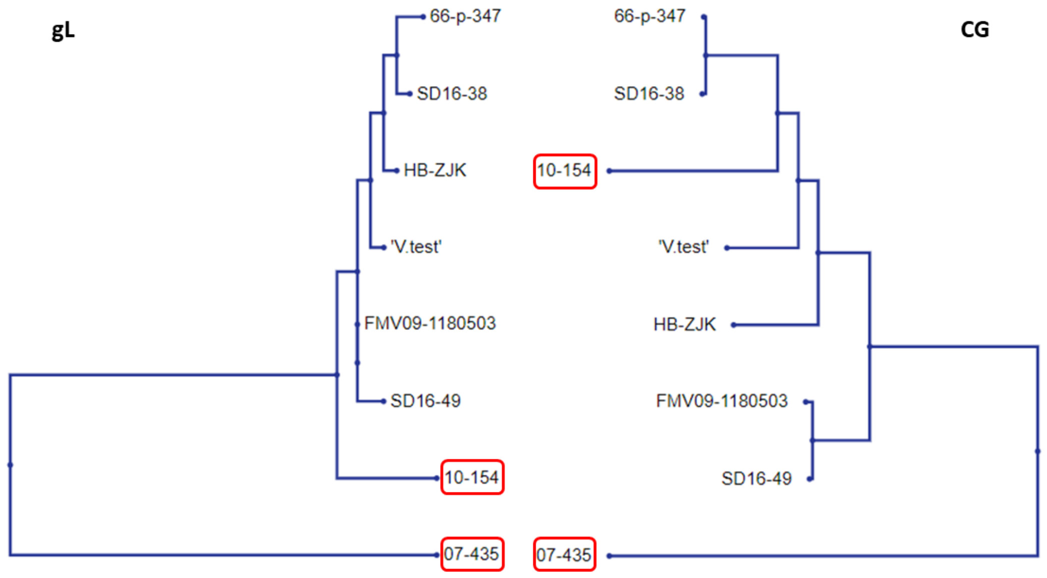

Figure S6. Comparison of phylogenetic trees obtained the Bovine Gammaherpesvirus 4 genome (right) and gL (left) sequences. Bovine Gammaherpesvirus 4 strains reported in the current study are highlighted by red boxes The trees were displayed using the Phylo.io application (Robinson et al., 2016) in the TreeCmp software v2.0 (Goluch et al., 2020).

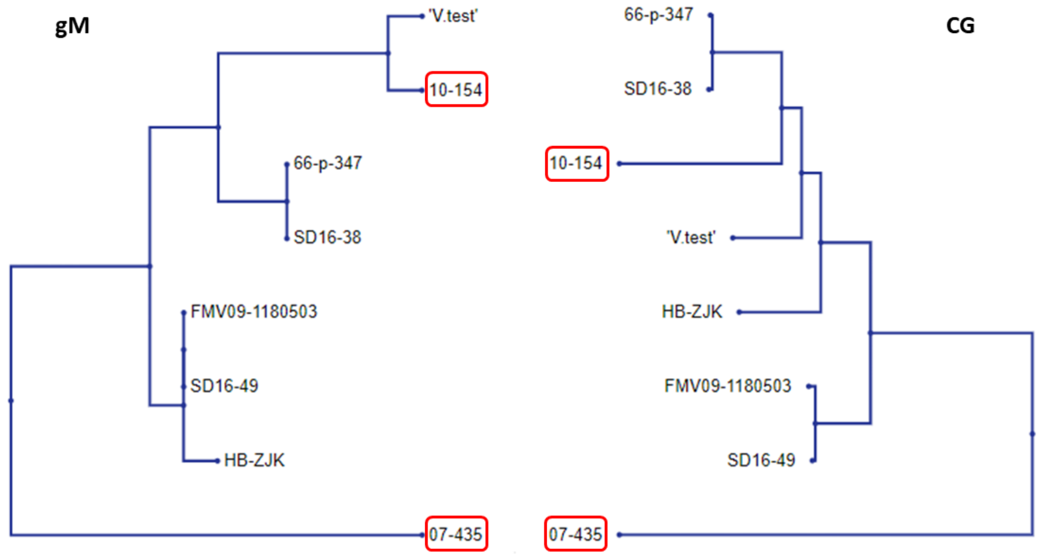

Figure S7. Comparison of phylogenetic trees obtained the bovine herpesvirus 4 genome (right) and gM (left) sequences. Bovine Gammaherpesvirus 4 strains reported in the current study are highlighted by red boxes. The trees were displayed using the Phylo.io application (Robinson et al., 2016) in the TreeCmp software v2.0 (Goluch et al., 2020).

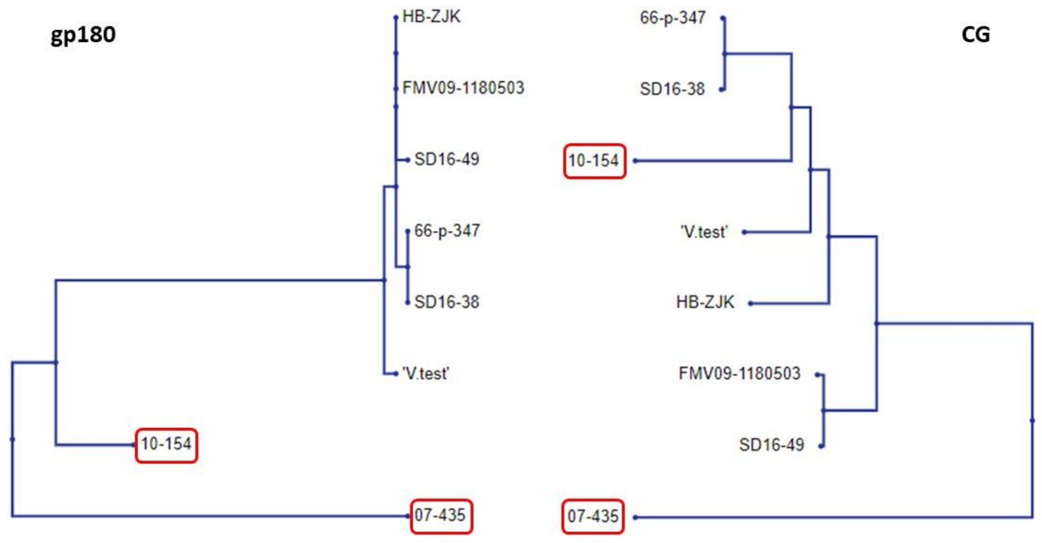

Figure S8. Comparison of phylogenetic trees obtained the Bovine Gammaherpesvirus 4 genome (right) and gp180 (left) sequences. Bovine Gammaherpesvirus 4 strains reported in the current study are highlighted by red boxes. The trees were displayed using the Phylo.io application (Robinson et al., 2016) in the TreeCmp software v2.0 (Goluch et al., 2020).

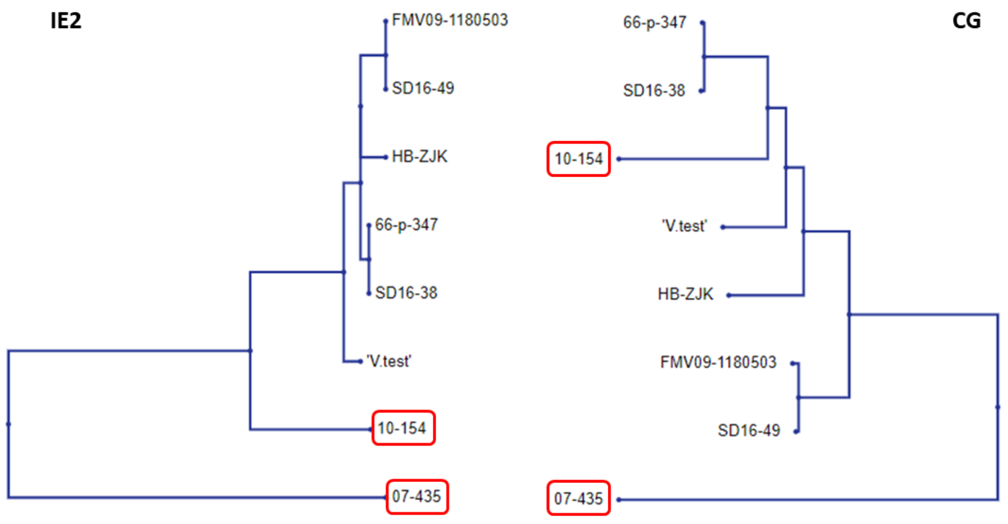

Figure S9. Comparison of phylogenetic trees obtained the Bovine Gammaherpesvirus 4 genome (right) and IE2 (left) sequences. Bovine Gammaherpesvirus 4 strains reported in the current study are highlighted by red boxes. The trees were displayed using the Phylo.io application (Robinson et al., 2016) in the TreeCmp software v2.0 (Goluch et al., 2020).

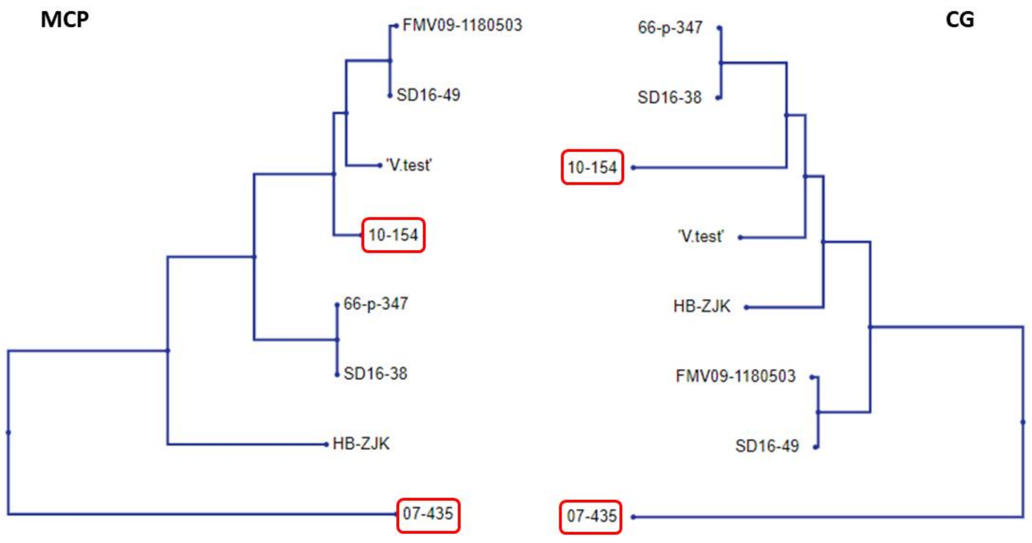

Figure S10. Comparison of phylogenetic trees obtained the Bovine Gammaherpesvirus 4 genome (right) and MCP (left) sequences. Bovine Gammaherpesvirus 4 strains reported in the current study are highlighted by red boxes. The trees were displayed using the Phylo.io application (Robinson et al., 2016) in the TreeCmp software v2.0 (Goluch et al., 2020).

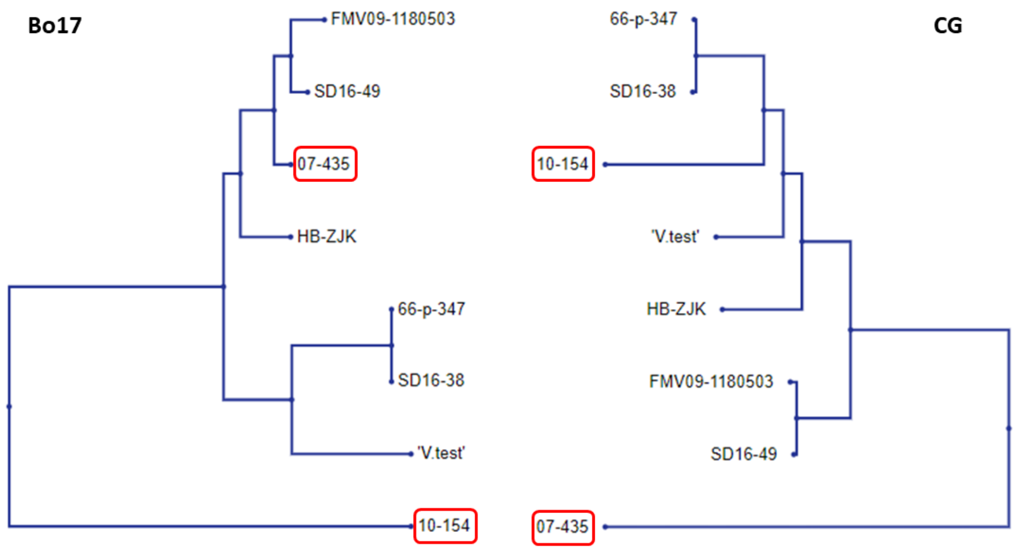

Figure S11. Comparison of phylogenetic trees obtained the Bovine Gammaherpesvirus 4 genome (right) and Bo17 (left) sequences. Bovine Gammaherpesvirus 4 strains reported in the current study are highlighted by red boxes. The trees were displayed using the Phylo.io application (Robinson et al., 2016) in the TreeCmp software v2.0 (Goluch et al., 2020).

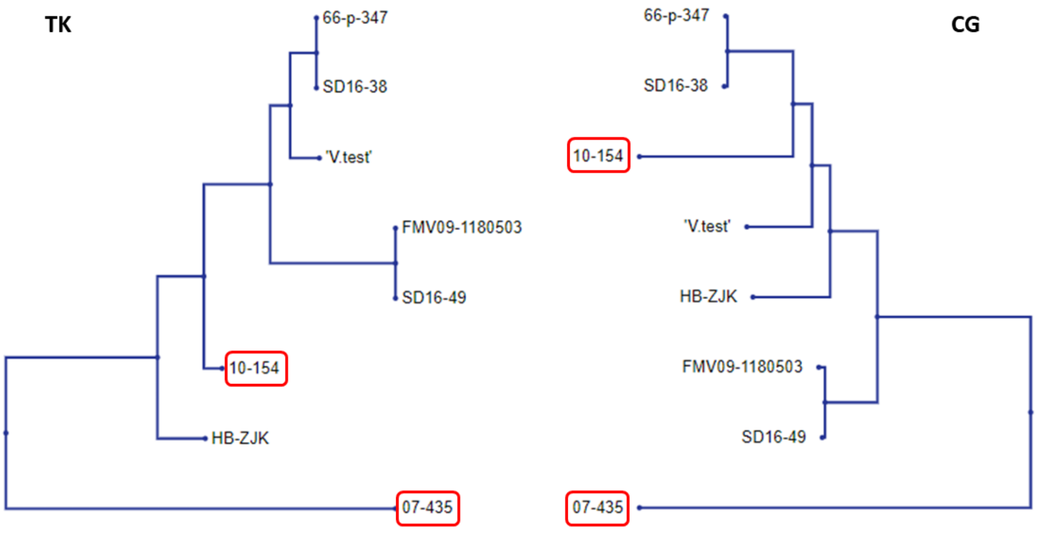

Figure S12. Comparison of phylogenetic trees obtained the Bovine Gammaherpesvirus 4 genome (right) and TK (left) sequences. Bovine Gammaherpesvirus 4 strains reported in the current study are highlighted by red boxes. The trees were displayed using the Phylo.io application (Robinson et al., 2016) in the TreeCmp software v2.0 (Goluch et al., 2020).
